# Supplementary material for: Intravital imaging and cavitation monitoring of antivascular ultrasound in tumor microvasculature
Source: Theranostics. 2023 Jan 1;13(1):250–66. doi: 10.7150/thno.79186 (PMC9800738; doi:10.7150/thno.79186)
Supplement: Supplementary file 1 — Supplementary figures and tables. [file thnov13p0250s1.pdf]

Supplementary Information

Table S1. Antivascular ultrasound preclinical studies. *An overview of animal models, cavitation agents, ultrasound setups, and outcomes in preclinical antivascular ultrasound microbubble studies. (* = combination with chemotherapy, ** = combination with immunotherapy, *** = combination with radiation therapy, † = brain study)*

Reference	Model	Cavitation Agent	Ultrasound Setup	Key Results
Wood *et al.*, UMB 2005 [85]	C3H/HeN mice<br><br>Murine melanoma (K1735)<br><br>Subcutaneous	Optison™<br><br>Bolus of 0.1 mL	D150 Plus, Dynatronics Corp. physiotherapy device<br><br>1 MHz, I_{SATA} = 2.3 W/cm^2, CW for 1, 2, or 3 min<br><br>Imaging with contrast-enhanced power Doppler immediately after and 24 h after sonication	**Assessments: (acute) histology, perfusion**<br><br>Each minute of sonication led to a 25% reduction in tumour vascularity which persisted for 24 h<br><br>Histology demonstrated disruption of vascular walls and tumour cell death in areas of vascular congestion and thrombosis
Hwang *et al.*, UMB 2005 [16]	White rabbit<br><br>Auricular vein	Optison™<br><br>Bolus of 7.3x10^7 MB/kg<br><br>7x Optison™ clinical imaging dose	Focused transducer (APC 880, APC International Ltd.) - 34.9 mm diameter, 5 cm focal length<br><br>1.17 MHz, 500 cycle pulse length, 5 Hz PRF, for 1 min<br><br>P = 1, 3.3, 6.5, or 9 MPa	**Assessments: (acute) histology**<br><br>Significant endothelial damage in larger vessels (~1 mm diameter) on luminal surface<br><br>No thermal damage to perivascular tissues
Hwang *et al.*, UMB 2006 [11]	White rabbit<br><br>Auricular vein	Optison™<br><br>Bolus of 7.3x10^7 MB/kg<br><br>7x Optison™ clinical imaging dose	Focused transducer (APC 880, APC International Ltd.) - 34.9 mm diameter, 5 cm focal length<br><br>1.17 MHz, 500 or 5000 cycle pulse length, 1 Hz PRF, for 1-120 s<br><br>P = 1, 3, 6.5, or 9 MPa	**Assessments: (acute) histology**<br><br>Significant endothelial damage, platelet adhesion to endothelial surface, and formation of intravascular fibrin thrombus, with damage increasing with pressure<br><br>Mechanical rather than thermal injury
Wood *et al.*, ISTU 2006 [86]	C3H/HeN mice<br><br>Murine melanoma (K1735)<br><br>Subcutaneous	Optison™<br><br>Bolus of 0.1 mL	D150 Plus, Dynatronics Corp. physiotherapy device<br><br>1 MHz, I_{SATA} = 2.3 W/cm^2, CW for 1, 2, or 3 min<br><br>Imaging with contrast-enhanced power Doppler immediately after and 24 h after sonication	**Assessments: (acute) histology, perfusion**<br><br>Decreased perfusion with increasing treatment time<br><br>Tumour vessels preferentially affected compared to mature, larger, and healthy vessels<br><br>Necrosis of neoplastic cells
Wood *et al.*, Acad Radiol 2008 [87]	C3H/HeN mice<br><br>Murine melanoma (K1735)<br><br>Subcutaneous	Definity™<br><br>Bolus of 8.5x10^{10} MB/kg<br><br>3400x Definity™ clinical imaging dose	D150 Plus, Dynatronics Corp. physiotherapy device<br><br>1 or 3 MHz, 2.4 W/cm^2, CW for 3 min<br><br>P = 0.27 MPa<br><br>Imaging with B-mode and contrast-enhanced power Doppler (7-15 MHz probe, HDI 5000 SonoCT, Philips)	**Assessments: (acute) histology, perfusion, temperature**<br><br>Reduction in tumour perfusion by 75% at 3 MHz (enhanced perfusion reduction at 3 MHz compared to 1 MHz), measured at 24 h<br><br>Predominant effects: dilation of capillaries, hemorrhage<br><br>Temperature increase of 7.8°C at 1 MHz, 15°C at 3 MHz
Chin *et al.*, IEEE IUS Proc 2009 [61]	C57BL6 mice<br><br>Murine colon adenocarcinoma (MC38)<br><br>Subcutaneous	In-house lipid MBs with C_4F_{10} core (mean diameter 2 μm)<br><br>2 bolus injections of 25x10^8 MB/kg, separated by 10 min<br><br>100x Definity™ clinical imaging dose	TIPS device (Philips)<br><br>1.2 MHz, 3 pulse trains of 10 pulses of 100000 cycles at 1 Hz PRF, separated by 20 s off period)<br><br>P = 5 MPa<br><br>Treatment applied after each of the 2 MB bolus injections<br><br>Imaging with HDI5000 or Sonos 7500 (Philips) scanners with CL15-7 or 15-6L probes	**Assessments: (acute) perfusion, temperature, (longitudinal) tumour volume, survival**<br><br>No temperature rise during treatment<br><br>Acute blood flow disruption after single treatment, with flow returning after 5-10 min<br><br>Blunted tumour growth with ultrasound-stimulated MB treatment
Wood *et al.*, UMB 2010 [34]	C3H/HeN mice<br><br>Murine melanoma (K1735)<br><br>Subcutaneous	Definity™<br><br>Bolus of 8.5x10^{10} MB/kg<br><br>3400x Definity™ clinical imaging dose	D150 Plus, Dynatronics Corp. physiotherapy device<br><br>3 MHz, 2.4 W/cm^2, CW for 1 min, x3 (with 1 min between treatments)<br><br>P = 0.27 MPa<br><br>Tumour volume measurements with B-mode ultrasound (7-15 MHz probe, HDI 5000 SonoCT, Philips)	**Assessments: (longitudinal) tumour volume, survival**<br><br>Reduction in tumour growth rate, and increased survival time

Burke *et al.,* J Neurosurg 2011 [88]	C57BL6/Rag-1 mice<br><br>Glioma (C6)<br><br>Subcutaneous	In-house albumin MBs with C_3F_8 gas core (~2 µm mean diameter)<br><br>Infusion of 10^8 MB/kg (continuously throughout experiment)<br><br>1.5x Definity™ clinical imaging dose	Unfocused transducer (A314S, Panametrics) – 19.05 mm diameter<br><br>1 MHz, 5000 or 10000 cycle pulse length, x5 bursts separated by 50 ms (duty cycle 0.5 or 1%). Repeated every 5 s for 1 h.<br><br>P = 1-1.2 MPa<br><br>Imaging with Cadence Contrast Pulse Sequencing (8-15 MHz 15L8 probe, Acuson Sequoia 512, Siemens)	**Assessments: (acute) histology, perfusion, temperature**<br><br>Duty-cycle dependent blood flow reduction immediately after treatment (Post-treatment perfused area down to 4% of pre-treatment at 1% duty-cycle)<br><br>Duty-cycle dependent increase in intratumoural temperature (increase of 2.5°C at 0.5% duty-cycle, 5°C at 1% duty-cycle)<br><br>Significant increase in tumour cell necrosis and apoptosis after treatment
Liu *et al.,* J Transl Med 2012 [89]	BALB/c mice<br><br>Murine colon carcinoma (CT26)<br><br>Subcutaneous	SonoVue®<br><br>Bolus of 0.1 mL/kg	Focused transducer (Sonic Concepts) – 64 mm diameter, 55 mm focal length<br><br>0.5 MHz, 100 ms pulse length, 1 Hz PRF, for 20 s (9-12 sonications to cover the full tumour)<br><br>P = 0.6 or 1.4 MPa	**Assessments: (acute) histology, flow cytometry, temperature, (longitudinal) tumour volume, survival**<br><br>2 h post-treatment demonstrated a significant increase in vessel permeability, enlarged extracellular spaces with local red blood cell extravasation, but no increase yet in apoptotic cells<br><br>Local modulation of immune cells (transient increase in non-Treg tumor infiltrating lymphocytes, continual infiltration of CD8+ cytotoxic T-lymphocytes, increased CD8+/Treg ratio)<br><br>No temperature increase<br><br>Decrease in tumour volume after 16 days (18% at 0.6 MPa, 34% at 1.4 MPa)
Hu *et al.,* Invest Radiol 2012 [32]	FVB mice<br><br>Murine mammary carcinoma (Met-1 or NDL)<br><br>Orthotopic (mammary fat pad)	Visistar® Integrin cRGD-conjugated MBs (Targeson)<br><br>In-house LXY-3 peptide-conjugated MBs<br><br>In-house non-targeted, non-biotinylated MBs<br><br>Mean diameter 2 µm (all)<br><br>Bolus of $5x10^9$ MB/kg	Sequoia 512, Siemens, with a 15L8 probe (7 MHz centre frequency)<br><br>5 MHz colour Doppler pulses, 6 cycles, 124 Hz PRF, 900 ms duration<br><br>P = 2 or 4 MPa<br><br>Treatment performed 7 min after MB injection (for clearance of untargeted agent)<br><br>Imaging with Cadence Contrast Pulse Sequencing at 230 kPa, 10 Hz frame rate	**Assessments: (acute) histology, perfusion**<br><br>Reduced perfusion after treatment, to a greater extent with higher pressure<br><br>Vasculature recovery within 40 min<br><br>Observed vasodilation and enhanced extravasation of injected dextran<br><br>Pre-treatment administration of anti-CD41 antibody prevented reduction in tumour blood flow, pointing to a mechanism of platelet activation<br><br>Greater therapeutic effect in Met-1 tumours than NDL tumours
Huang *et al.,* Oncotarget 2015 [65]	BALB/c nude mice<br><br>Human pancreatic cancer (XPA-1)<br><br>Subcutaneous	Targestar®-P (Targeson) – lipid-encapsulated C_4F_{10} MB (mean diameter: 1.9 or 2.9 µm)<br><br>Bolus of $5x10^9$ MB/kg (retro-orbital)	$1cm^2$ tip sonicator (Haiying Medical Electronic Instrument Co.)<br><br>238 kHz, 10 ms pulse length, duty-cycle 50%, for 1 min (repeated over 3 successive days)<br><br>P = 0.5 MPa<br><br>Contrast-enhanced ultrasound imaging on day 1 prior to treatment and day 3 after final treatment (4-11 MHz LA332 probe, Mylab90 scanner)	**Assessments: (acute) histology, perfusion, (longitudinal) whole-body fluorescence for tumour volume**<br><br>Ultrasound treatment with both MB sizes resulted in a decrease in tumour perfusion 3 days post-treatment<br><br>Significant tumour cell apoptosis with treatment, more significant with larger MBs<br><br>Both MB sizes with ultrasound treatment blunted tumour growth, with the larger MBs having greater efficacy by day 24
Yang *et al.,* Onc Lett 2015 [90]	BALB/c nude mice<br><br>Human prostate adenocarcinoma (PC3)<br><br>Subcutaneous	Albumin MB with C_3F_8 core (Kangrui Pharmaceutical Co.), mean diameter 3.4 µm<br><br>Bolus, doses of 0.05, 0.1, or 0.2 mL at $6.5x10^8$ MB/mL (equivalent to $1.6\text{-}6.5x10^9$ MB/kg)	3 different low frequency ultrasound systems (Shanghai Institute of Ultrasound in Medicine)<br><br>20, 80, or 500 kHz, duty-cycle of 20% (1 s on, 4 s off), 40% (2 s on, 3 s off), or 60% (3 s on, 2 s off), for 1, 3, or 5 min<br><br>Intensity = 0.5, 1, or 2 W/cm^2 *(intensity type, i.e.* I_{spta} *or* I_{sppa}*, not indicated)*	**Assessments: (acute) histology, perfusion**<br><br>Vessel wall disruption, vasodilation, edema, greater effects with increasing pressure<br><br>All parameters tested influenced perfusion, with the optimal being 20 Hz, 1 W/cm^2, duty-cycle 40%, 3 min, MB dose 0.2 mL

			Contrast-enhanced ultrasound imaging (4-11 MHz LA332 probe, Mylab90 scanner)	
Wang *et al.,* IJC 2015 [35]	Kunming mice<br><br>Murine sarcoma (S180)<br><br>Subcutaneous	Lipid-shelled C_3F_8 MBs<br><br>Bolus of $7.5x10^9$ MB/kg	KHT-017 pulsed therapeutic transducer (DCT-700, Shenzhen Well.D Medical Electronics Co.)<br><br>0.94 MHz, 0.19% duty cycle, 10 Hz PRF, 3 s on and 9 s off for 1 min<br><br>P = 0.5, 1.5, 3, or 5 MPa<br><br>B-mode and contrast-enhanced ultrasound imaging (15L8 probe, Sequoia 512, Siemens)	**Assessments: (acute) histology, perfusion, (longitudinal) tumour volume, survival**<br><br>Tumour cell necrosis and apoptosis more prevalent with increasing pressure<br><br>Perfusion reduction at all pressures (measured immediately and 24h after US), more sustained at higher pressures (84% decrease at 24 h after treatment at 3 MPa)<br><br>Extreme reduction in number of immature vessels in tumour, while decrease in mature vessels was not significant<br><br>Delayed tumour growth and increased survival with increasing pressure
Hunt *et al.,* JUM 2015 [42]	C3H/HeN mice<br><br>Murine melanoma (K1735)<br><br>Subcutaneous	Definity™<br><br>Bolus of $8.5x10^{10}$ MB/kg	D150 Plus, Dynatronics Corp. physiotherapy device (unfocused)<br><br>3 MHz, 2.3 W/cm^2, CW for 1 or 3 min<br><br>P = 0.22 MPa<br><br>Power Doppler contrast-enhanced imaging	**Assessments: (acute) histology, perfusion, flow cytometry**<br><br>Treatment time-dependent decrease in perfusion, with a 45% decrease after 1 min, 67% after 3 min (perfusion assessed immediately and 5 h after treatment)<br><br>Hemorrhages in regions of decreased perfusion, as well as dilated and thrombosed vessels<br><br>Local inflammatory response (more HIF1A+ cells and CD45+CD3+ T cell infiltration)
Keravnou *et al.,* UMB 2016 [62]	Pig liver (*ex vivo*, machine perfused)	In-house lipid-MBs with a C_4F_{10} core (mean diameter 2.5 μm)<br><br>Bolus (2 mL) of $3\text{-}8x10^6$ MB/mL (every 1 min)	Focused, single-element transducer – 60 mm diameter, 75 mm focal length<br><br>1 MHz, 20 or 1000 cycles, duty-cycle 2 or 8%<br><br>Ultrasound 5 s on, 5 s off for every MB bolus, repeated in same location for 15 min<br><br>P = 1.7, 2.5, 4 MPa<br><br>Imaging with iU22 Philips diagnostic scanner	**Assessments: (acute) perfusion**<br><br>Detectable and irreversible perfusion changes above 1.7 MPa, with complete devascularization at 4 MPa<br><br>Less perfusion changes with shorter pulses compared to longer pulses at the same pressure
Ho *et al.,* Drug Discov Today 2017 [51]	C57BL6/JNarl mice<br><br>Transgenic adenocarcinoma murine prostate (TRAMP)<br><br>Subcutaneous (dorsal window chamber)	Lipid-based MBs vs. nanodroplets (ND) vs. microdroplets (MD)<br><br>Dose of $5x10^7$ particles/mouse	2 MHz focused transducer, 1.2 mm focal diameter, single 3 cycle pulse<br><br>P = 3, 5, 7, 10 MPa	**Assessments: (acute) extravasation from microvessels**<br><br>Dye release is inversely proportional to vessel size and proportional to pressure<br><br>At 10 MPa:<br>MBs: extravasation from < 40 μm vessels<br>MD: extravasation from < 50 μm vessels<br>ND: extravasation from < 30 μm vessels<br><br>Maximum disrupted vessel size:<br>MBs: 20 μm at 3 MPa, 40 μm at 5 and 10 MPa<br>MD: 50 μm at 10 MPa<br>ND: 30 μm at 10 MPa
Ho *et al.,* ACS Appl Mater Interfaces 2018 [53]	C57BL6/JNarl mice<br><br>Transgenic adenocarcinoma murine prostate (TRAMP)<br><br>Subcutaneous (dorsal window chamber and hind limb solid tumour)	Lipid-based MBs (mean diameter 1.12 μm)<br><br>Dose of $1x10^7$ MB/mL<br><br>Solid tumour treatment groups: Lipo-Dox (5 mg/kg), Lipo-Dox + 7 MPa, MBs + 5 MPa, MBs + 7 MPa, Lipo-Dox + MBs + 7 MPa<br><br>Perfusion assessment with SonoVue®	Therapy with a 2 MHz focused transducer (SU-101, Sonic Concepts)<br><br>P = 1, 3, 5, 7, 9 MPa (3 cycles) or P = 7 MPa (3, 50, or 100 cycles) in 'normal' dorsal window chamber tissue for microscopy<br><br>P = 5, 7 MPa (1000 cycles) in subcutaneous tumours<br><br>B-mode perfusion imaging with a 7 MHz ultrasound imaging system (Terason t3000)	**Assessments: (acute) extravasation from microvessels, histology, perfusion, (longitudinal) tumour growth, survival**<br><br>Increasing vessel size disruption and extravasation distance with pressure (3 cycles), no change as a function of number of cycles (7 MPa)<br><br>Blood flow reduction in tumour core that does not recover over 10 days, greater with increasing pressure in combination with MBs<br><br>Enhanced survival with MBs + 5 MPa, MBs + 7 MPa, Lipo-Dox + MBs + 7 MPa (in increasing order)

Sun *et al.,* Appl Acoust 2018 [54]	New Zealand white rabbits<br><br>Leporine anaplastic squamous cell carcinoma (VX2)<br><br>Orthotopic (liver)	SonoVue ®<br><br>Bolus of 0.6 mL of 2x10^8 MB/mL	Low frequency focused ultrasound therapy apparatus (Institute of Technology Innovation Company of Zhejiang University); multidimensional movement structure with a concave disk ultrasonic transducer (1x1x3 cm^3 focus)<br><br>370 kHz, 22.5 min total exposure with 3 consecutive daily cavitation treatments: 9 treatment points to cover the tumour, each point treated 15x for a total of 150 s with a 10% duty cycle (MB bolus at 0, 50, 100 s of treatment)<br><br>P = 1.5 MPa<br><br>Contrast-enhanced ultrasound imaging with Mylab90 (Esaote) with a LA322 linear array probe (3-11 MHz, MI 0.04) at 1, 7, 14, 21 days after treatment	**Assessments: (acute) histology, perfusion, (longitudinal) tumour growth**<br><br>Tumours treated with US+MBs exhibited a significant decrease in perfusion in the core for at least 7 days (some recovery after 14 days)<br><br>Observed thrombosis in treated tumours via histology
Jing *et al.,* J Canc Res Clin Onc 2019 [91]	Nude mice<br><br>Human breast carcinoma (MDA-MB-231)<br><br>Subcutaneous	In-house unconjugated lipid MBs (2.9 µm) and Endostar-MBs (2.8 µm)<br><br>Drug content: 800.72 µg/10^8 MBs	838A-H-O-S ultrasound treatment device (Shengxiang Ultrasonic)<br><br>840 kHz, 10 s pulse length, 10 s interval, for 2 min<br><br>Intensity = 0.75 W/cm^2 *(intensity type, i.e.* I_{spta} *or* I_{sppa}*, not indicated)*<br><br>Imaging (30 min later) with SonoVue® and Aplio500 (Toshiba) with a PLT-805AT probe at 8 MHz	**Assessments: (acute) histology, flow cytometry**<br><br>Endostar-MBs significantly enhanced drug delivery compared to untargeted MBs<br><br>Combination group also significantly decreased microvessel density and lowered VEGF expression levels
Yemane *et al.,* UMB 2019 [63]	BALB/c nude mice<br><br>Human osteosarcoma (OHS)<br><br>Subcutaneous (dorsal window chamber)	In-house nanoparticle-stabilized MBs (NP-MBs; 2.4 µm diameter)<br><br>SonoVue ® (2.5 µm diameter)<br><br>Concentration matched bolus of 2-5x10^8 MB/mL	Single-element focused transducer – 60 mm diameter, 75 mm curvature<br><br>1 MHz, 10 ms pulse length, 0.5 or 1 Hz PRF, 5 min<br><br>P = 0.2, 0.4, 0.6, or 0.8 MPa	**Assessments: (acute) extravasation from microvessels, perfusion changes**<br><br>Extravasation at 0.5 Hz PRF higher for NP-MBs than for SonoVue® at 0.8 MPa (73% vs. 44%) and 0.4 MPa (56% vs. 22%)<br><br>More extravasation at lower PRF of 0.1 Hz compared to 0.5 Hz<br><br>80% of extravasation events occurred at vessel branching points<br><br>Blood flow speed changes during ultrasound exposure, with greater decreases in speed with increasing pressure: NPs decreased speed by 41% (0.2 MPa), 63% (0.4 MPa), 89% (0.8 MPa) with NP-MBs, and by ~70% with SonoVue®<br><br>Blood flow direction changes increased in prevalence with increasing pressure. At the highest pressure, 50% of recordings revealed a change in flow direction
D'Souza *et al.*, Nanotheranostics 2019 [57]	Wistar rats<br><br>Hepatocellular carcinoma (HCC)<br><br>Orthotopic (liver)	Definity™<br><br>Primary regimen: Bolus of 0.5 mL prior to first insonation and 0.2 mL prior to second insonation<br><br>Reduced dose regimen: Bolus of 0.1 mL prior to each insonation	D150 Plus, Dynatronics Corp. physiotherapy device<br><br>Primary regimen: 3 MHz, 2 W/cm^2, CW 3x 2 min insonations separated by 2 min<br><br>Reduced dose regimen: 3 MHz, 1 W/cm^2, CW 3x 1 min insonations separated by 1 min<br><br>B-mode and contrast-enhanced ultrasound imaging (VisualSonics, VevoLAZR, 21 MHz linear transducer at 18 MHz, power = 4, contrast gain = 41, 2D gain = 18, sensitivity = 3), and power Doppler (16 MHz, 4-6 fps, 37 dB gain, power = 100%)	**Assessments: (acute) histology, perfusion**<br><br>Substantial decrease in tumour perfusion after primary regimen (peak enhancement in nonlinear contrast scans showed a 37.9% decrease)<br><br>Reduced dose regimen did not significantly change perfusion parameters
He *et al.,* Front Pharm 2020 [92]	Rabbits	In-house Zhifuxian MBs (C_3F_8 encapsulated in	KHT-017 pulsed therapeutic transducer (DCT-700, Shenzhen	**Assessments: (acute) histology, perfusion, (longitudinal) tumour volume**

	Leporine anaplastic squamous cell carcinoma (VX2)<br><br>Intramuscular	lipid shell, mean diameter 2 μm)<br><br>6-9x10^9 MB/mL, ultrasound therapy dose of 0.1 mL/kg<br><br>Infusion of 5 mL suspension at 1.25 mL/min	Well.D Medical Electronics Co.), unfocused<br><br>1 MHz, 10 Hz PRF, intermittent (9 s on, 3 s off) for 5 min<br><br>P = 1, 2, 3, 4, or 5 MPa<br><br>B-mode and contrast-enhanced ultrasound imaging (iU22, Philips, with L12-5 probe)	More extensive hemorrhage with increasing pressure, with severely ruptured microvessels and microvascular debris at 4-5 MPa<br><br>Gradual reduction in blood perfusion in tumour with increasing pressure from 2-5 MPa (measured immediately after US)<br><br>At 3, 4 MPa – perfusion completely blocked in tumour centre (periphery intact)<br>At 5 MPa – perfusion blocked in tumour centre and surrounding muscle<br><br>Both single and multiple treatment sessions led to a reduction in tumour growth
Todorova *et al.,* IJC 2012 [30]<br><br>*	Athymic mice<br><br>Human breast carcinoma (MDA-MB-231)<br><br>Subcutaneous	Definity™<br><br>Bolus of 60 μL/kg (5x10^8 MB/kg), x3 in 10 min intervals<br><br>20x Definity™ clinical imaging dose	1 MHz, single-element focused transducer, short bursts (0.00024 duty cycle)<br><br>P = 1.6 MPa<br><br>Weekly ultrasound treatments for 4 weeks, with metronomic cyclophosphamide chemotherapy for 10 weeks<br><br>Cavitation recording (passive) with single-element 750 kHz, focal length 7.5 cm, diameter 2.5 cm<br><br>Imaging with Toshiba Aplio (7 MHz probe in contrast imaging mode at MI 0.05, 11 Hz frame rate)	**Assessments: (acute) histology, perfusion, cavitation, (longitudinal) tumour volume, survival**<br><br>Acute reduction of perfusion (monitored immediately after treatment), sustained 24 h and 3 days post-treatment<br><br>Cavitation monitoring revealed inertial cavitation, sub-, and ultra-harmonic peaks<br><br>Significant tumour growth inhibition with USMB treatment, with significantly more growth inhibition and extended survival when combined with chemotherapy<br><br>Rationale for combining chemotherapy with ultrasound mechanical ablation is that they act on different tumour regions; chemotherapy suppresses mobilization and tumour recruitment of bone marrow-derived cells induced by mechanical ablation
Goertz *et al*, PLoS One 2012 [15]<br><br>*	Athymic mice<br><br>Human prostate adenocarcinoma (PC3)<br><br>Subcutaneous	Artenga MBs (C3F8 core in a Span 60 / Tween 80 shell, mean diameter 2.13 μm)<br><br>Bolus of 2.1x10^8 MB/kg, equivalent to 40 μL/kg Definity™<br><br>Groups: MB, DTX (docetaxel), USMB, USMB+DTX	1 MHz, single-element focused transducer, 50 ms pulse length, short bursts (0.00024 duty cycle)<br><br>P = 1.6 MPa<br><br>Cavitation recording (passive) with single-element 750 kHz, focal length 7.5 cm, diameter 2.5 cm	**Assessments: (acute) histology, perfusion, cavitation, (longitudinal) tumour volume, survival**<br><br>Acute reduction of perfusion (monitored immediately after treatment), significant 10-fold reduction of flow in central region (not periphery) with USMB+DTX treatment<br><br>Histology revealed higher levels of necrosis and apoptosis with USMB+DTX treatment<br><br>Cavitation monitoring revealed inertial cavitation, sub-, and ultra-harmonic peaks<br><br>Combination group yielded significant tumour growth delay and improved survival
Keller *et al.,* Front Pharm 2020 [93]<br><br>*	Pten$^{fl/fl}$;Alb^{cre} (*Pten*-null) mice<br><br>Naturally developed liver tumours similar to human HCCs	SonoVue®<br><br>Bolus of 50 uL at 1-5x10^8 MB/mL x4 injections<br><br>Groups: 30 mg/kg doxorubicin (DOX) with or without USMBs	S5-1 phased array on an EPIQ scanner (Philips), in hybrid pulsed-wave Doppler mode<br><br>1.6 MHz, focal length of 10 cm, 200 cycles, 50 Hz PRF<br><br>4 injections with treatment 30 s after injection, alternating 5 s on, 5 s off for 30 s total on time (90 s between injections)<br><br>P = 2-3 MPa *(exact pressure unknown)*<br><br>B-mode and contrast-enhanced ultrasound imaging (iU22, Philips, with L12-5 probe)	**Assessments: (acute) histology, perfusion**<br><br>DOX + USMB treatments resulted in a high degree of immediate antivascular action selectively within tumours<br><br>Significant (~two-fold) increase in dox accumulation in tumours of mice with USMBs
Bulner *et al*, UMB 2019 [41]<br><br>**	BALB/c mice<br><br>Murine colon carcinoma (CT26)<br><br>Subcutaneous	Artenga MBs (C3F8 core in a Span 60 / Tween 80 shell, number mean diameter 1.1 μm, volume mean diameter 3.7 μm)<br><br>Bolus of 50 μL, dose of 9.6x10^8 MB/kg	1 MHz transducer (Valpey Fisher, 3.75 cm diameter, 15 cm focus), 0.1 ms pulses spaced 1 ms apart x50, repeated at 20 s intervals for 2 min<br><br>P = 1.65 MPa	**Assessments: (acute) histology, perfusion, flow cytometry, cavitation, (longitudinal) tumour volume, survival**<br><br>Immediate perfusion shutdown visualized, and histology indicated higher levels of necrosis and apoptosis with combination treatment

		Groups: MB, aPD-1, USMB, USMB+aPD01	Cavitation recording (passive) with single-element 750 kHz, focal length 7.5 cm, diameter 2.5 cm<br><br>Contrast imaging (L12-5 probe, EPIQ 7G, Philips) at MI 0.07, 11 Hz frame rate	Cavitation monitoring revealed inertial cavitation, sub-, and ultra-harmonic peaks<br><br>Combination group yielded significantly enhanced tumour growth inhibition and survival<br><br>Flow cytometry and enzyme-linked immunospot analysis did not clearly support a T cell-dependent mechanism
Czarnota *et al,* PNAS 2012 [47]<br><br>***	CB-17 severe compromised immunodeficient (SCID) mice<br><br>Human prostate adenocarcinoma (PC3)<br><br>Subcutaneous	Definity™<br><br>Low dose of 3.6x10^8 MB or high dose of 1.08x10^9 MB (100- and 300-fold higher than diagnostic dose, respectively)<br><br>Radiation therapy (RT) of 0, 2, or 8 Gy combined with USMBs<br><br>Targeted MB experiments with avidin-conjugated MicroMarker Target-Ready agent (VisualSonics), with biotinylated VEGFR2 antibody (equivalent to low concentration experiments)	500 kHz focused transducer (IL0509HP Valpey Fisher), 28.6 mm diameter, 16 cycle bursts, 3 kHz PRF, 10% duty cycle, 50 ms duration, repeated every 2 s for a total of 5 min<br><br>P = 570 kPa<br><br>Power Doppler imaging for perfusion monitoring (0, 3, 6, 12, 24 h)	**Assessments: (acute) histology, perfusion**<br><br>USMB+RT induces an over 10-fold greater cell kill, and enables a much lower radiation dose for comparable effect without USMB treatment<br><br>Even greater effect of USMB+RT when targeted MBs were used<br><br>Induction of ceramide-related endothelial cell apoptosis leading to vascular disruption is a causative mechanism
El Kaffas *et al,* PLoS One 2014 [64]<br><br>**, ***	Athymic nude mice<br><br>Colon adenocarcinoma (LS174T)<br><br>Subcutaneous	Definity™<br><br>Dose of 3% v/v (volume %), or 1.08x10^9 MB in 90 µL, equivalent to 300-fold diagnostic dose<br><br>Groups: Control, XRT, Dll4 mAb, XRT+Dll4 mAb, USMB+XRT, USMB+XRT+Dll4 mAb	500 kHz focused transducer (IL0509HP Valpey Fisher), 28.6 mm diameter, 16 cycle bursts, 3 kHz PRF, 10% duty cycle, 50 ms duration, repeated every 2 s for a total of 5 min<br><br>P = 570 kPa<br><br>Power Doppler imaging for perfusion monitoring (24 h, 7 days)	**Assessments: (acute) perfusion, histology, (longitudinal) perfusion**<br><br>Dll4 mAb maintained the shutdown achieved by USMB+RT<br><br>Vascular shutdown persisted at least 7 days in mice with triple combination treatment
Lai *et al,* OncoScience 2016 [44]<br><br>***	Swiss nude mice<br><br>Human breast carcinoma (MDA-MB-231)<br><br>Subcutaneous	Definity™<br><br>Dose of 3% v/v, or 1.08x10^9 MB in 90 µL, equivalent to 300-fold diagnostic dose<br><br>Radiation therapy (RT) of 0, 2, or 8 Gy combined with USMBs	500 kHz focused transducer (IL0509HP Valpey Fisher), 28.6 mm diameter, 16 cycle bursts, 3 kHz PRF, 10% duty cycle, 50 ms duration, repeated every 2 s for a total of 5 min<br><br>P = 570 kPa	**Assessments: (acute) histology, (longitudinal) tumour volume**<br><br>Tumour cell apoptosis, vascular leakage, decrease in tumour vasculature, delay in tumour growth – synergistic effect with USMB+RT<br><br>Damage to microvasculature regulates tumour cell response to radiation<br><br>Endothelial cell perturbation leading to activation of gene expression pathways, stimulated by radiation
Daecher *et al,* Cancer Letters 2017 [43]<br><br>***	Immunodeficient nude rats<br><br>Hepatocellular carcinoma (HCC)<br><br>Orthotopic, liver	Optison™<br><br>Infusion of 0.1 mL over 10-20 s, 2.4x10^8 MB/kg<br><br>22x Optison™ clinical imaging dose<br><br>Combined with 5 Gy RT	4.2 MHz, 1.6 µs pulses, 38 Hz PRF transmitted in a series of 4 s destructive pulses at MI 1.35, then followed by 10 s lower intensity CPS imaging at MI 0.06 to monitor perfusion (treatment duration 2-3 min)<br><br>Siemens S2000 scanner, 9L4 probe<br><br>P = 2.5 MPa	**Assessments: (acute) perfusion, (longitudinal) tumour volume, survival**<br><br>Reduction in tumour vascularity with ultrasound treatment, with a further (linear) reduction with subsequent destructive pulses (67% decrease after 3 pulses)<br><br>Significant improvement in survival time for combination therapy (RT 5 Gy single dose, 3 h post-USMB)
Al-Mahrouki *et al*, PLoS One 2017 [45]<br><br>***	CB-17 severe compromised immunodeficient (SCID) mice<br><br>Human prostate adenocarcinoma (PC3)<br><br>Subcutaneous	Definity™<br><br>Dose of 6x10^8 MB *in vitro*, or 3% v/v (1.08x10^9 MB in 90 µL, equivalent to 300-fold diagnostic dose) *in vivo*<br><br>Groups: USMB, RT (8 Gy), USMB+RT (8 Gy)	500 kHz transducer (ValpeyFisher), bursts repeated every 2 s for 5 min total<br><br>*In vitro:* 240 kPa, 10% duty cycle, 30 s<br>*In vivo:* 570 kPa, 10% duty cycle, 16 cycle bursts, 3 kHz PRF, 50 ms duration<br><br>For combination treatments, RT given shortly after USMB with Faxitron X-ray cabinet at 200 cGy/min	**Assessments: (acute) histology, perfusion, oxygen saturation levels**<br><br>Combination treatment results in highest levels of apoptosis, vascular disruption, and lowest oxygen saturation<br><br>When UGT8 levels are reduced, ceramide accumulates or initiates apoptotic signal

			Power Doppler (Vevo770, VisualSonics) and photoacoustic imaging (Vevo2100, VisualSonics)	
El Kaffas *et al,* Theranostics 2018 [46]<br><br>***	C57BL6 mice<br><br>Fibrosarcoma (MCA-129)<br><br>Subcutaneous	Definity™<br><br>1% v/v (25 µL in 75 µL saline) or 3% v/v (70 µL in 30 µL saline)<br><br>Radiation therapy (RT) of 0, 2, or 8 Gy combined with USMB	500 kHz focused transducer (IL0509HP Valpey Fisher), 28.6 mm diameter, 16 cycle bursts, 3 kHz PRF, 10% duty cycle, 50 ms duration, repeated every 2 s for a total of 5 min<br><br>P = 570 kPa	**Assessments: (acute) histology, perfusion**<br><br>USMBs had no significant effects on tumour perfusion, microvascular density, ISEL, ceramide (3, 24, 72 h) alone, but after RT (single dose 2 or 8 Gy), there was significant acute reduction in blood flow<br><br>Combination USMB+RT (8 Gy) results in 50% decrease in tumour perfusion, peaking at 24h and persisting for up to 72 h, accompanied by tumour cell apoptosis and necrosis
El Kaffas *et al,* J Nat Canc Inst 2018 [94]<br><br>***	Wild type or ASMase knockout mice<br><br>Fibrosarcoma (MCA-129)<br><br>Subcutaneous	Definity™<br><br>1% v/v ($1x10^{10}$ MB/kg) or 3% v/v ($3x10^{10}$ MB/kg), equivalent to 71x or 205x Definity™ clinical dose<br><br>Radiation therapy (RT) of 0, 2, or 8 Gy combined with USMBs	500 kHz focused transducer (IL0509HP Valpey Fisher), 28.6 mm diameter, 16 cycle bursts, 3 kHz PRF, 5 min<br><br>P = 570 kPa<br><br>3D Doppler ultrasound for perfusion monitoring	**Assessments: (acute) histology, perfusion, (longitudinal) tumour volume**<br><br>No effect on tumour perfusion with USMB treatment only (at either dose), but combination with RT resulted in a decrease of up to 46.5% at 3 h (peaked at 24 h, persisted up to 72 h)<br><br>Stunted tumour growth with combination treatment<br><br>Proposed mechanism of mechanodestructive vascular targeting of ASMase-ceramide pathway
McDannold *et al*, Radiology 2006 [95]<br><br>†	Rabbits<br><br>Normal brain	Optison™<br><br>Bolus of 0.05 mL/kg, or $3.65x10^{7}$ MB/kg<br><br>3.6x Optison™ clinical dose	1.5 MHz focused transducer, 10 cm diameter, 8 cm focal length, 500 ms pulse length, 1 Hz PRF, for 10 or 20 s<br><br>P = 2-4 MPa	**Assessments: (acute) histology, MRI**<br><br>Necrotic lesions created with time-averaged power much less than what would be required for thermal lesions (without MBs)
McDannold *et al,* J Neurosurg 2013 [36]<br><br>†	Wistar rats<br><br>Normal brain	Definity™<br><br>Bolus of 10 or 20 µL/kg	525 kHz spherically curved, air-backed transducer (4 cm diameter, 3 cm radius of curvature), 10 ms bursts, 1 Hz PRF, 5 min<br><br>P = 174 or 195 kPa	**Assessments: (acute) histology, MRI**<br><br>Destruction of vasculature produced lesions and ischemia in downstream tissues<br><br>Damage limited to endothelium, preferentially affected more vascularized gray matter
Huang *et al,* UMB 2013 [96]<br><br>†	Sprague-Dawley rats<br><br>Normal brain	Definity™<br><br>Bolus of 0.02 mL/kg<br><br>10x Definity™ clinical imaging dose	558 kHz, 10 cm diameter, 7.8 cm radius of curvature, 10 ms bursts, 1 Hz PRF, 5 min<br><br>At some locations, 2 ms or longer bursts (200 ms, 500 ms, or CW) of 5 min or 1 min sonications were tested<br><br>P = 267, 300, 366, or 444 kPa	**Assessments: (acute) histology, MRI**<br><br>Probability of lesion production (2-48 h), with lesions being predominantly ischemic necrosis: 267 kPa = 39%, 300 kPa = 50%, 366 kPa = 61%, 444 kPa = 71%<br><br>BBBD and edema evident with MRI imaging<br><br>Shorter, 1 min sonications: unlikely to produce necrotic lesions, only scattered red blood cell leakage and selective neuronal necrosis<br><br>Shorter bursts (2 ms) had lower probability of inducing lesions, while longer bursts (200 ms) caused destruction of vessel walls and mechanical destruction of brain parenchyma. At 500 ms or CW, shielding prevented damage
Arvanitis *et al*, J Neurosurg 2016 [66]<br><br>†	Rhesus macaques<br><br>Normal brain	Definity™<br><br>Bolus of 20 µL/kg (2x clinical imaging dose)<br><br>Infusion at 0.1 mL/min for 10 s, then 0.02 mL/min	ExAblate 4000 low-frequency TcMRgFUS system (InSightec), 30 cm diameter, hemispherical 1024 element phased array<br><br>220 kHz, 10 ms bursts, 1 Hz PRF, 5 min<br><br>P = 500 kPa<br><br>Cavitation recording with 2 weakly focused (15 cm radius), air-backed, rectangular (4x0.7 cm) PZT transducers at 610 kHz mounted on each side of animal's head, 10 cm from focus of array	**Assessments: (acute) histology, MRI, cavitation monitoring**<br><br>Bolus: strong inertial cavitation at start for 10 s, then low-level broadband activity<br><br>Infusion: sporadic strong inertial cavitation, strength of low-level broadband activity increased over time<br><br>When inertial cavitation was present, histology and MRI revealed localized ischemic necrosis, lesions with central region having hemorrhage and edema<br><br>Non-thermal lesions created, with blood-brain barrier disruption in lesions and prefocal area of FUS system

McDannold *et al,* J Neurosurg 2016 [97]<br><br>†	Sprague-Dawley rats<br><br>Normal brain	Optison™<br><br>Bolus of 100 µL/kg, or $7.3x10^7$ MB/kg<br><br>6.6x Optison™ clinical imaging dose	1.1 MHz focused transducer, 10 ms bursts, 1 Hz PRF, 5 min<br><br>P = 0.8 MPa	**Assessments: (acute) histology, MRI, (longitudinal) MRI**<br><br>Hemorrhages and lesions evident immediately after sonication, with some white matter areas remaining partially intact<br><br>Cystic lesions formed within 2 weeks of treatment
Jones *et al,* Theranostics 2020 [67]<br><br>†	Rabbits<br><br>Normal brain	Definity™<br><br>Infusion of 0.2 mL/kg over 90 s<br><br>10x Definity™ clinical imaging dose	In-house multifrequency transmit/receive sparse hemispherical phased array system (256 modules distributed over a 31.8 cm diameter aperture, each module with 3 concentric cylindrical PZT-4 elements)<br><br>612 kHz, 10 ms bursts, 1 Hz PRF, 2 min<br><br>Pressure ramp (0.2-0.25 MPa, with 30-40 kPa steps), step size reduced by a factor of 2 following first subharmonic detection event<br><br>Subharmonic thresholds of 0, 50, 100, or 150% of pressure	**Assessments: (acute) histology, MRI, cavitation**<br><br>Tissue damage volumes increased with increasing exposure levels<br><br>Histology revealed small zones of red blood cell leakage and overt tissue damage from exposures at 50% and 0% of the subharmonic pressure threshold that were not evident on T2*w MRI<br><br>Ultrafast MB imaging provided superior predictive capability than that obtained with conventional temporal average processing

Table S2. Vessel sampling data. *Mean and standard deviation of sampled vessel diameters at each pressure, as well as the mean vessel diameters exhibiting focal disruption or flow shutdown events.*

Tissue Type	Pressure	n Subjects	n Vessels	Vessels (µm)	Focal Disruption (µm)	Flow Shutdown (µm)
Tumour	1 MPa	7	464	11.1 ± 8.0	8.0 ± 4.9	8.7 ± 4.0
	2 MPa	9	693	10.0 ± 13.4	7.2 ± 5.2	8.2 ± 4.9
	3 MPa	7	464	12.0 ± 14.5	11.2 ± 13.4	11.1 ± 9.2

Table S3. Shutdown in small vs. large vessels. *Of the vessels that experienced shutdown, transient shutdown (i.e. recovered), and sustained shutdown, the following table outlines relative incidence in small (< 20 µm) vs. large (> 20 µm) vessels.*

Tissue Type	Pressure	n Subjects	% Shutdown		% Recovered		% Sustained	
			< 20µm	> 20µm	< 20µm	> 20µm	< 20µm	> 20µm
Tumour	1 MPa	7	100.0	0.0	100.0	0.0	100.0	0.0
	2 MPa	9	97.5	2.5	97.4	2.6	97.6	2.4
	3 MPa	7	89.8	10.2	90.9	9.1	89.8	10.2
Healthy	1 MPa	3	100.0	0.0	100.0	0.0	0.0	0.0
	2 MPa	4	88.9	11.1	100.0	0.0	75.0	25.0
	3 MPa	4	92.9	7.1	100.0	0.0	90.6	9.4

Supplementary Videos: See accompanying .avi files for Videos S1-8.

Video S1. *Immediately upon sonication, a strong vascular event appears to occur outside of the FOV (in a deeper plane) along with a bulk vasospasm event in the same region (lower right area). Throughout the rest of the sonication, vascular deformation (or vasospasm) continues throughout the FOV (indicated by arrows), also resulting in flow alterations and sometimes brief cessation (indicated by asterisks). (Normal tissue; 2 MPa)*

Video S2. *Shortly after the start of sonication (10 s), violent vascular deformation (indicated by arrows) and focal disruptions (encircled regions) occur throughout the FOV, followed by flow shutdown (denoted by asterisks). A clot (denoted by an X) also forms within a vessel near the largest focal disruption. (Tumour tissue; 3 MPa)*

Video S3. *Shortly after the start of sonication (10 s), the second vessel from the left undergoes vasoconstriction (from 14 – 24 s), indicated by a line denoting the original diameter. Blood flow in the constricted vessel slows and appears to shutdown, evidenced by the vessel turning dark. Nearby vessels also undergo flow changes (denoted by asterisks), with the ones in the left possibly being affected by the vasoconstricted vessel (though events may have occurred outside the FOV), and the ones on the right being affected by focal disruptions (encircled regions appearing to diffuse to this plane of depth throughout the scan). A clot (denoted by an X) also forms in the large vessel on the right side of the FOV, partially occluding the vessel and causing flow changes. (Tumour tissue; 2 MPa)*

Video S4. *Shortly after initiating sonication, focal disruption events (encircled regions) occur in the vessels on the left in the FOV. A clot forms at one of the disruption points (denoted by an X), and another disruption occurs nearby at a bifurcation. A vessel in the middle near the top of the FOV undergoes constriction and then dilation (indicated by a line denoting the original diameter), and several nearby nodes also then yield focal disruption events. (Tumour tissue; 2 MPa)*

Video S5. *Immediately upon sonication, several focal disruptions (encircled regions) occur at bifurcations in this complex tumour-affected microvascular network, followed by irregular flow (denoted by asterisks) that results in additional focal disruption events at 140 s. (Tumour tissue; 2 MPa)*

Video S6. *Several focal disruption (encircled regions) and corresponding local dilation events (with likely bubble explosion events and loss of vessel wall integrity) occur, followed by rapid blood flow shutdown (flow changes marked by asterisks) in most vessels. Flow begins to recover in some of the shutdown vessels with vasodilation evident at the end of the sonication (~3 min, 10 s). (Tumour tissue; 3 MPa)*

Video S7. *In this scan, slight vascular deformation is apparent (indicated by arrows), along with altered blood flow directionality (denoted by asterisks) throughout the network towards the end of the scan. (Tumour tissue; 1 MPa)*

Video S8. *A focal disruption event (encircled regions) occurring in the large vessel in the middle and towards the bottom of the FOV results in chaotically altered blood flow directionality and speed (denoted by asterisks) which appears to result in the aggregation of erythrocytes to form a large clot (indicated by an X). On the right, leakage events from just outside the FOV become apparent throughout the scan, as do flow alterations in nearby vessels. (Tumour tissue; 2 MPa)*

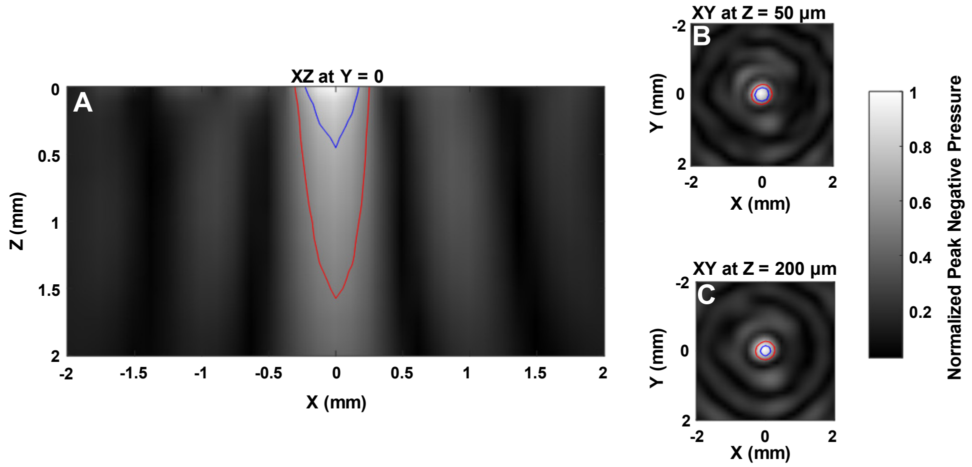

Figure S1. Ring transducer pressure profile. (A) *XZ pressure map, where Z = 0 is the location of the coverslip glued to the bottom of the transducer, and X = 0, Y = 0 is the centre of the ring transducer.* ***(B)*** *XY pressure map at a depth of Z = 50 µm and* ***(C)*** *Z = 200 µm. Red contours indicate -6 dB, and blue contours indicate -3 dB of the peak negative pressure. The rings have an axial FWHM of 1.55 mm, and a lateral FWHM of 0.6 mm at Z = 50 µm depth.*

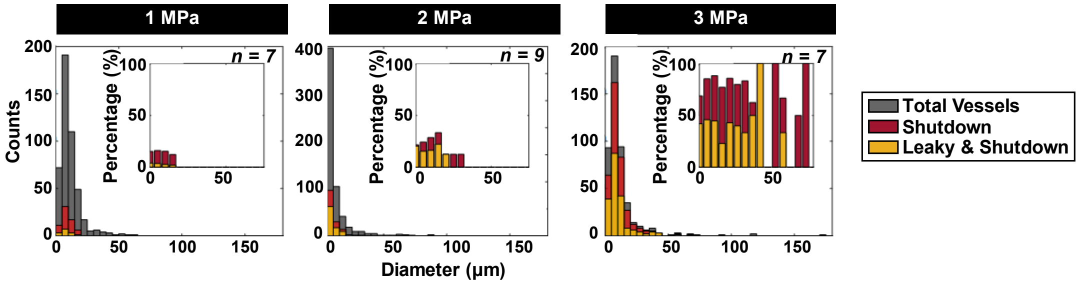

Figure S2. Shutdown and leakage event overlap. *Histograms of absolute counts of all visualized vessels overlayed by vessels exhibiting shutdown as well as both shutdown and leakiness at 1, 2, and 3 MPa in mice with tumours. Histograms of percentage of shutdown as well as shutdown and leaky vessels normalized to total visualized vessels are inset.*

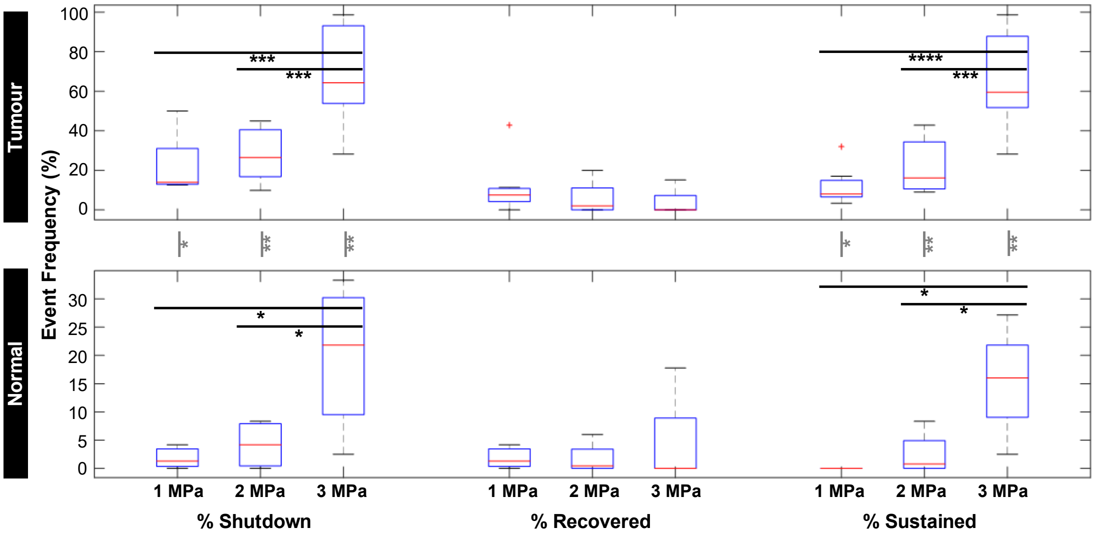

Figure S3. Shutdown (recovered and sustained) prevalence variability across mice. *Box and whisker plots displaying event frequency and variance of shutdown, recovered, and sustained vascular events across pressures and tissue type groups. The red crosshairs indicate outliers. p < 0.05 = *, p < 0.01 = **, p < 0.001 = ***, p < 0.0001 = ****.*

A

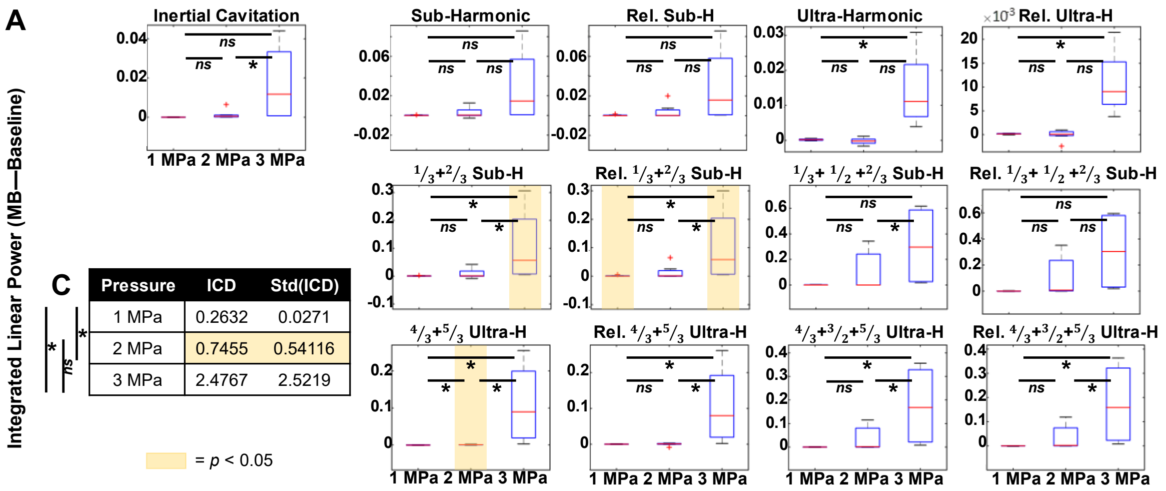

C

Pressure	ICD	Std(ICD)
1 MPa	0.2632	0.0271
2 MPa	0.7455	0.54116
3 MPa	2.4767	2.5219

B

Pressure	IC	Sub	Rel. Sub	1/3+2/3 Sub	Rel. 1/3+2/3 Sub	1/3+1/2+2/3 Sub	Rel. 1/3+1/2+2/3 Sub	Ultra	Rel. Ultra	4/3+ 5/3 Ultra	Rel. 4/3+ 5/3 Ultra	4/3+3/2+5/3 Ultra	Rel. 4/3+3/2+5/3 Ultra
1 MPa *(n = 5)*	0.821	0.149	0.168	0.102	0.024	0.120	0.155	0.105	0.098	0.559	0.180	0.098	0.064
2 MPa *(n = 7)*	0.091	0.127	0.104	0.759	0.583	0.128	0.100	0.083	0.081	0.022	0.570	0.080	0.078
3 MPa *(n = 4)*	0.100	0.122	0.118	0.046	0.032	0.115	0.112	0.077	0.075	0.076	0.082	0.073	0.073

Figure S4. Cavitation data summary for normal mice. (A) *Box and whisker plots displaying integrated power (of treatment with baseline subtracted) for each pressure group at various frequency peaks for the first burst in normal tissue.* ***(B)*** *Table of the mean power ratios for pressure groups at each frequency peak of interest. The red crosshairs indicate outliers. Groups with statistically significant (p < 0.05) elevation above baseline are highlighted in yellow.* ***(C)*** *IC dose (ICD) as a function of pressure. * = p < 0.05, n.s. = not significant.*

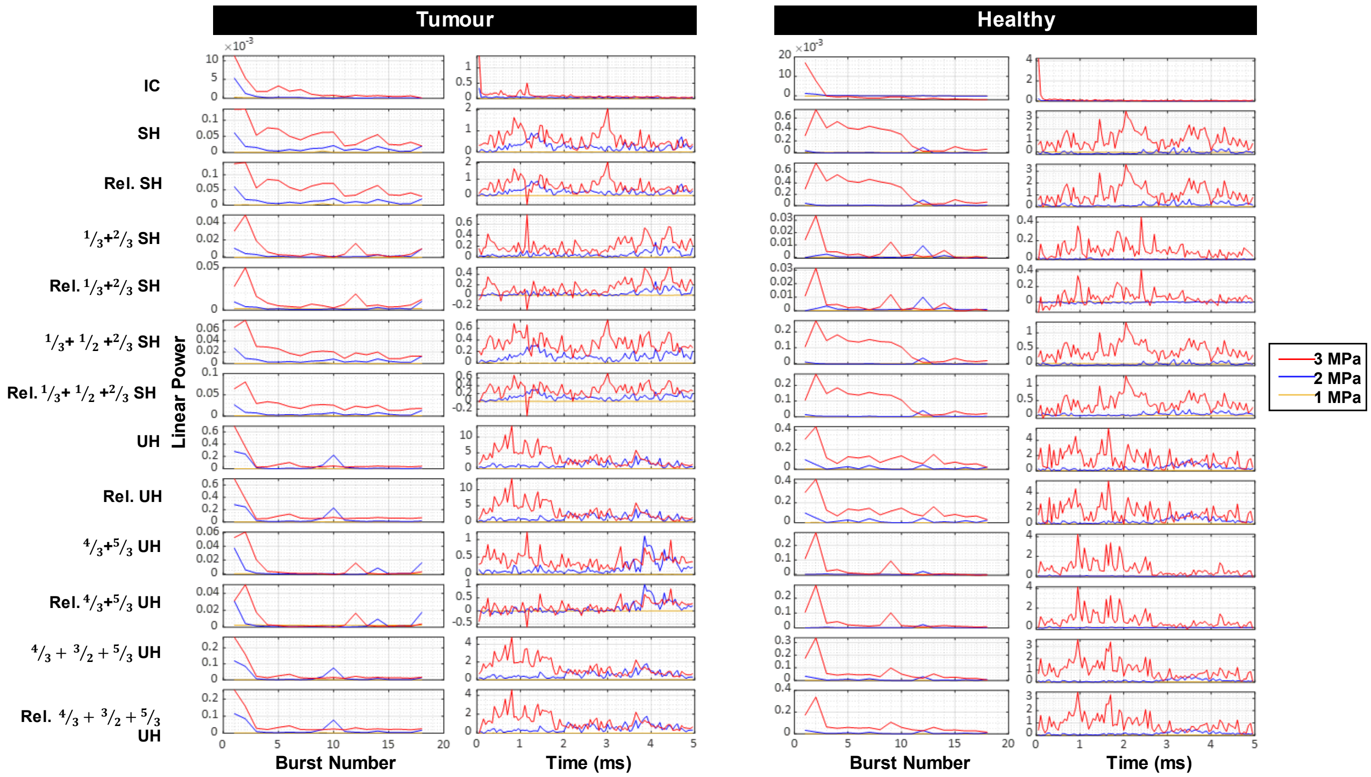

Figure S5. Persistence cavitation data for tumour and normal mice. *Traces of the average cavitation power over time in each frequency band of interest within the first burst and as a function of burst number at 1, 2, and 3 MPa.*
